# Supplementary figures and images for: Discovering differential genome sequence activity with interpretable and efficient deep learning
Source: PLoS Comput Biol. 2021 Aug 9;17(8):e1009282. doi: 10.1371/journal.pcbi.1009282 (PMC8376110; doi:10.1371/journal.pcbi.1009282)

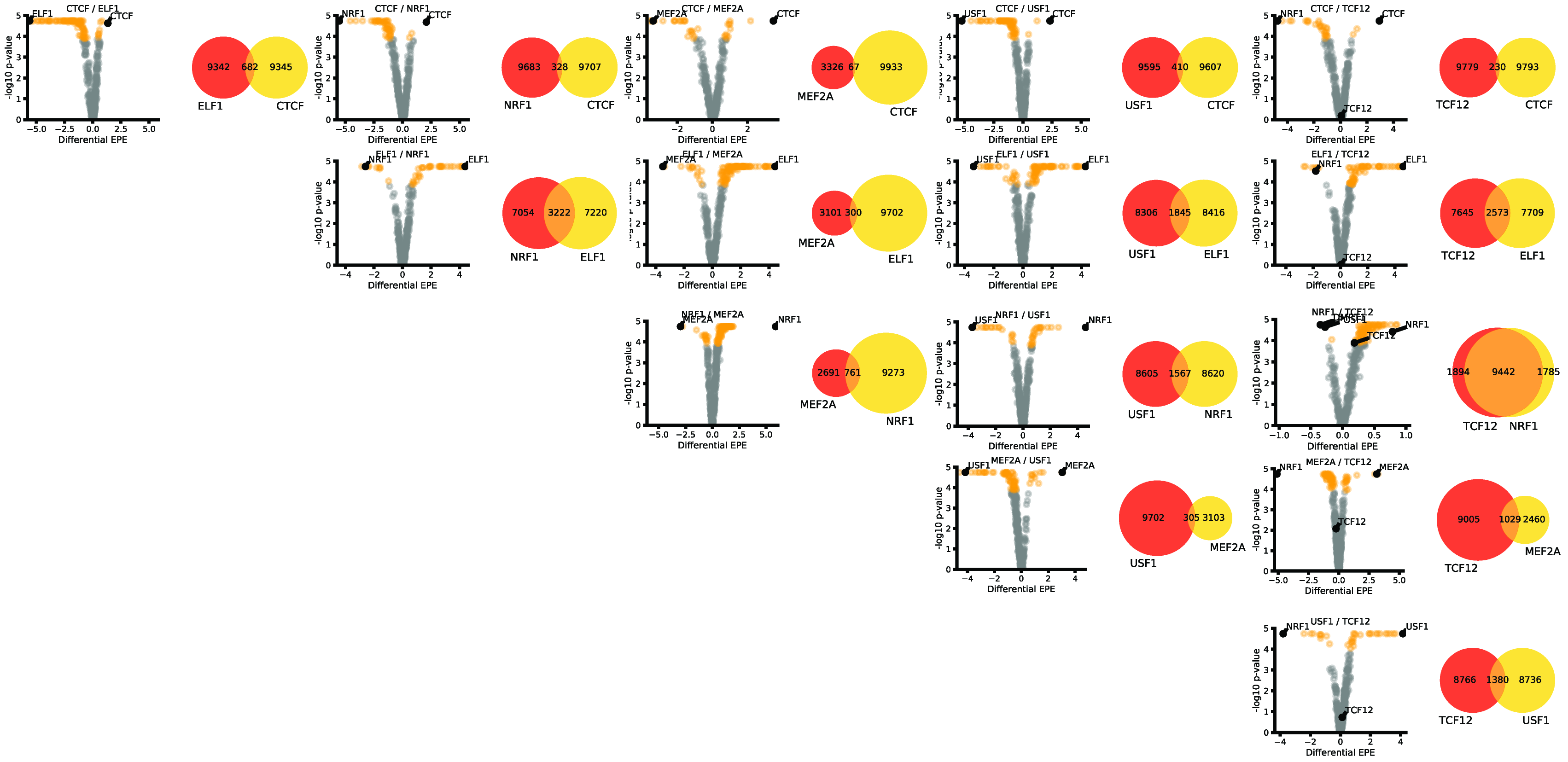

Supplement: S1 Fig — (TIF) [file pcbi.1009282.s003.tif]

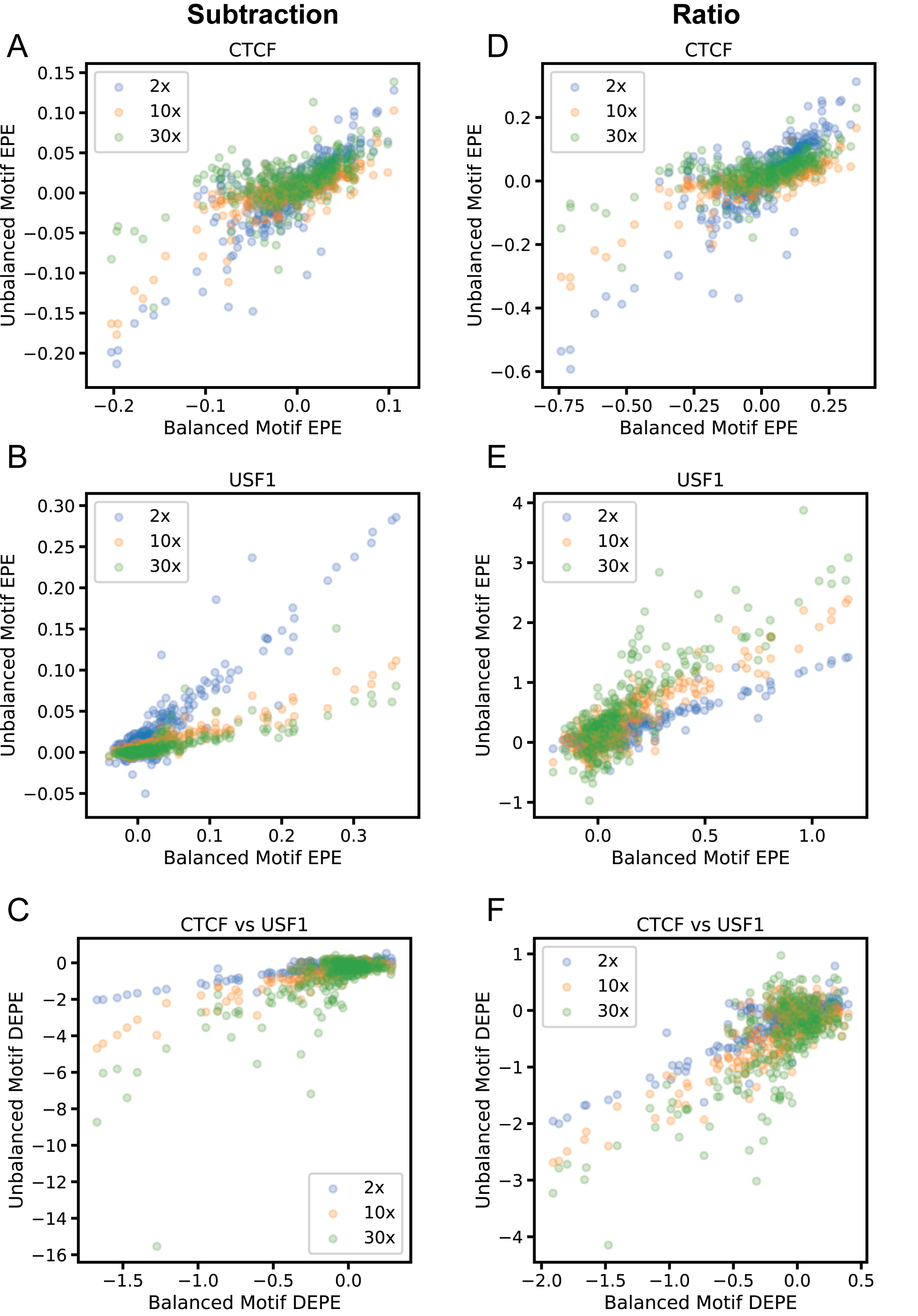

Supplement: S2 Fig — Scatter plots of GIA (A-C) or EPE/DEPE (D-F) of effect sizes of 356 HOCOMOCOv11 mouse motifs on predictions of CTCF binding from a trained DeepAccess model on balanced class data (x-axis) compared to 2x, 10x, and 30x more CTCF binding sites: USF1 binding sites (y-axis) (A) GIA of CTCF binding (B) GIA of USF1 binding (C) GIA of CTCF vs USF1 differential binding (D) EPE of CTCF binding (E) EPE of USF1 binding (F) DEPE of CTCF vs USF1 binding. (TIF) [file pcbi.1009282.s004.tif]

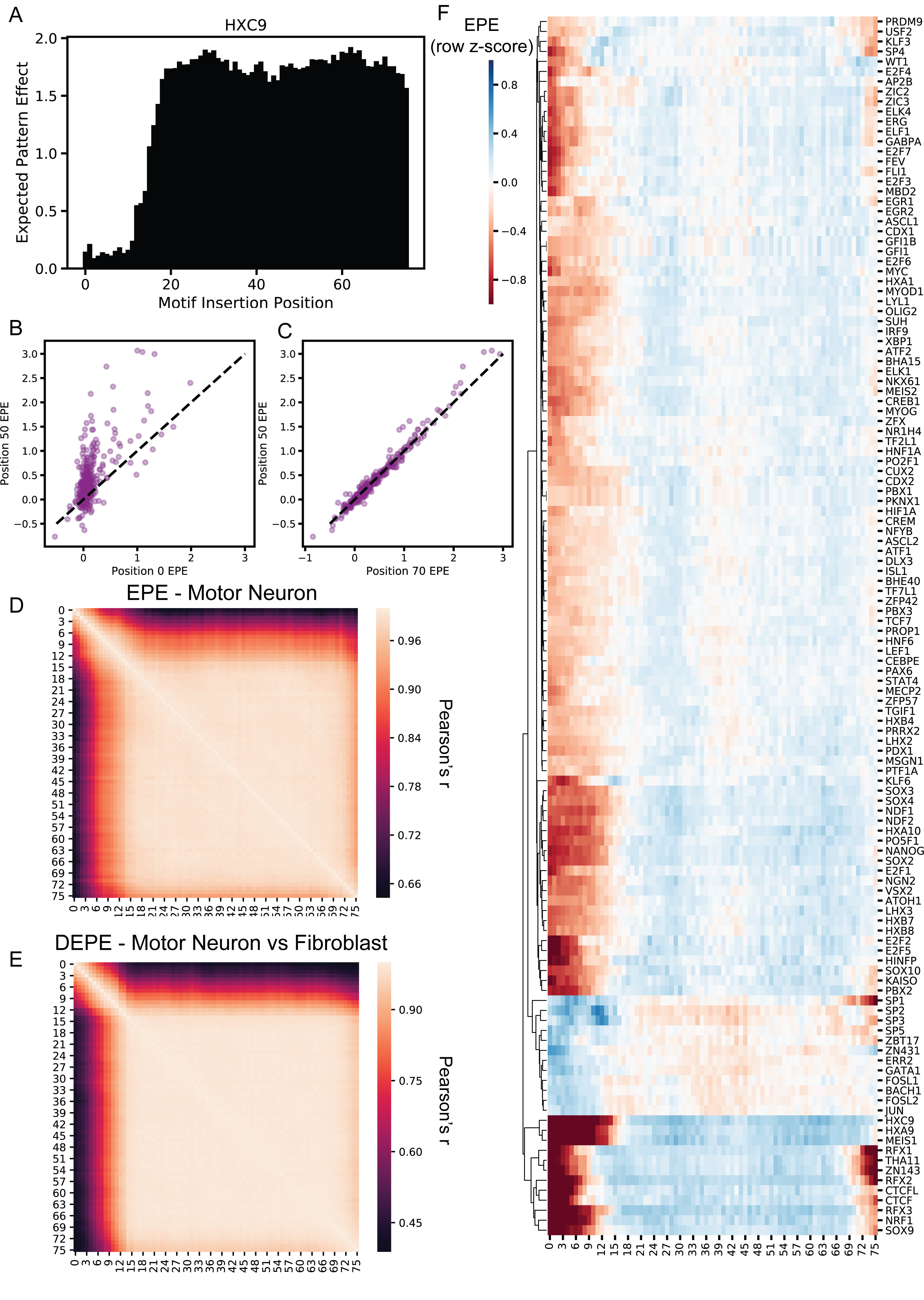

Supplement: S3 Fig — (A) EPE of Hoxc9 motif on EPE from DeepAccess model trained to predict motor neuron chromatin accessibility shows EPE of Hoxc9 motifs are low for positions in the beginning of the DNA sequence and high for positions at the middle and end of the DNA sequence. (B) Scatter plot of EPEs for 356 HOCOMOCOv11 mouse transcription factor motifs when insertion is at position 0 compared to position 50 indicate early insertion site in DNA sequence results in different EPE values for transcription factors, with some having lower EPEs and others having higher EPEs. (C) Scatter plot of EPEs for 356 HOCOMOCOv11 mouse transcription factor motifs when insertion is at position 70 compared to position 50 indicate early insertion site in DNA sequence results in similar EPE values for all transcription factors. (D) Heatmap of Pearson’s r of EPEs for DeepAccess motor neuron accessibility for insertions at all starting positions within the DNA sequence shows most insertion positions result in highly similar EPEs except for early insertion positions. (E) Heatmap of Pearson’s r of DEPEs for DeepAccess motor neuron vs fibroblast differential accessibility for insertions at all starting positions within the DNA sequence shows most insertion positions result in highly similar EPEs except for early insertion positions. (F) Row z-score normalized EPEs for DeepAccess motor neuron accessibility at each position show most motifs have higher EPEs when inserted at a position in the center of the DNA sequence. Some motifs (like Sp motifs) have higher EPEs at the beginning of the DNA sequence. (TIF) [file pcbi.1009282.s005.tif]

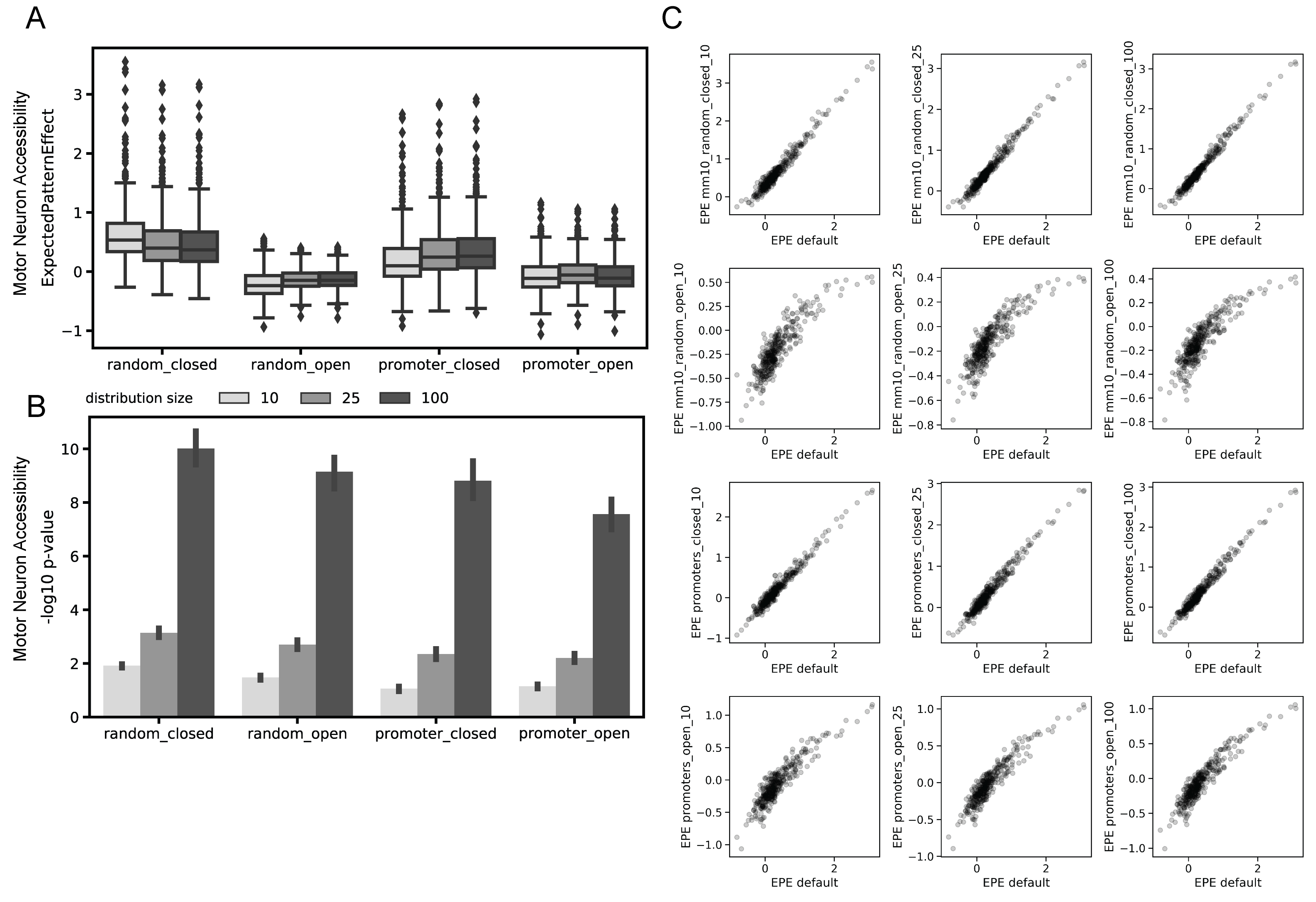

Supplement: S4 Fig — (A) Estimates of EPE for 356 HOCOMOCOv11 transcription factor motifs on predicted motor neuron accessibility from a learned DeepAccess model. M sequences are selected randomly from regions closed in all 10 cell types, open in all 10 cell types, promoters which are closed in all 10 cell types, and promoters which are open in all 10 cell types. Shade indicates distribution size (M = 10, M = 25, M = 100). Boxplot box indicates median and quartiles and whiskers extend to 1.5 times the inter-quartile range. Dots are outliers outside 1.5 times the inter-quartile range. (B) Estimates of significance of EPE for size and type of sequence used to estimate total sequence distribution. (C) Comparison of EPEs for 356 HOCOMOCOv11 transcription factor motifs for default (randomly selected 24 closed) compared to randomly selected closed (row 1), randomly selected open (row 2), promoter closed (row 3), and promoter open (row 4) shows while values of EPE dependent on selection of sequence distribution, rank remains relatively invariant. (TIF) [file pcbi.1009282.s006.tif]
